# Emerging Trends on the Correlation Between Neurotransmitters and Tumor Progression in the Last 20 Years: A Bibliometric Analysis *via* CiteSpace

**DOI:** 10.3389/fonc.2022.800499

**Published:** 2022-02-24

**Authors:** Yumiao Shi, Jiamei Luo, Xiaoqiang Wang, Yiqi Zhang, Hui Zhu, Diansan Su, Weifeng Yu, Jie Tian

**Affiliations:** Department of Anesthesiology, Renji Hospital, Shanghai Jiaotong University School of Medicine, Shanghai, China

**Keywords:** neurotransmitters, cancer, Citespace, bibliometric analysis, anesthesia and tumor

## Abstract

**Background:**

Bibliometric analysis is used to gain a systematic understanding of developments in the correlation between *neurotransmitters and tumor progression* in research hotspots over the past 20 years.

**Methods:**

Relevant publications from the Web of Science Core Collection (WoSCC) were downloaded on August 1, 2021. Acquired data were then analyzed using the Online Analysis Platform of Literature Metrology (http://biblimetric.com) and the CiteSpace software to analyze and predict trends and hot spots in this field.

**Results:**

A total of 1310 publications on *neurotransmitters and tumor progression* were identified, and 1285 qualified records were included in the final analysis. The country leading the research was the United States of America. The University of Buenos Aires featured the highest number of publications among all institutions. Co-citation cluster labels revealed the characteristics of 10 main clusters: beta-adrenergic receptors (β-AR), glutamate, neurotransmitters, serotonin, drd2, histamine, glycine, interleukin-2, neurokinin receptor-1, and nicotinic acetylcholine receptors (AchRs). Keywords and references burst detection indicated that apart from β-AR, dopamine receptor and cancer types like gastric cancer and glioblastoma are the newly emerging research hotspots.

**Conclusions:**

This study analyzed 1285 publications and 39677 references covering the topic of *neurotransmitters and tumor progression* and showed that while β-AR has always been a hot topic in this field, dopamine receptor is an emerging target for this research field, and gastric cancer and glioblastoma are the top two tumors that have garnered increasing attention and have become the focal point of recent studies.

## Introduction

Cancer has always been a major problem plaguing the health of the global population, and according to the latest data released by the World Health Organization in 2021, the total number of new patients with cancer worldwide in 2020 was about 19.29 million. In 2018, China had the highest number of tumor incidence and deaths across the world, which has brought a heavy medical burden to the country (1). Therefore, exploring more deeply the causes and finding effective cures for patients with cancer are clinically important.

Neurotransmitters are chemicals that transmit information between neurons and other types of cells, such as muscle and glandular cells. Based on their specific chemical structure, neurotransmitters are divided into four categories: 1) acetylcholine (Ach); 2) amino acids, including glutamate, aspartic acid, glycine, and gamma-aminobutyric acid (GABA); 3) biogenic amines, consisting of dopamine, norepinephrine (NE), epinephrine (E), and serotonin; and 4) neuropeptides, including but not limited to neuropeptide Y (NPY), neurotensin, and many others (2).

Neuromodulation in cancer is universal and involves complex mechanisms, which are not fully understood. As important messengers of neural signaling, neurotransmitters and their receptors contribute to tumor proliferation, angiogenesis and tumor metastasis (3–6). Additionally, neurotransmitter receptors are widely expressed on the surface of immune cells and regulated by their corresponding neurotransmitters, thus affecting tumor immune responses (7, 8).

For clinical anesthesia, the primary targets of many narcotics are various neurotransmitter receptors. For example, propofol is closely associated with GABA and N-methyl-D-aspartate (NMDA) receptors, and inhalational anesthetics, including sevoflurane desflurane, may mostly have a strong link with ionotropic neurotransmitter receptors. Muscle relaxants exert action through Ach receptors, whereas dexmedetomidine (Dex) mainly acts on α2-adrenergic receptors (α2-AR), among others. However, whether and how anesthetics affect tumor progression through neurotransmitter receptors remains unsettled.

Many authors worldwide have published research findings on neurotransmitters and tumor malignancy. Since many kinds of neurotransmitters are involved in the various types of cancer, the general direction of this body of research is challenging to grasp, and launching investigations in this field is difficult with little or no prior knowledge. Thus, collecting data from relevant publications is highly necessary to assist investigators in analyzing the vast amount of literature on this subject.

Bibliometric analysis is a method used to analyze large amounts of heterogeneous literature and is largely dependent on visualizing processing tools, like CiteSpace. The latter helps gather data on contributions to a certain field in diverse perspectives, including different countries/regions, institutions, journals, co-cited authors, co-cited networks, and detailed research trends or hot spots (9).

We aimed to provide a comprehensive understanding of the developments in the research on neurotransmitters and tumor progression by analyzing the remarkable achievements in the past 20 years. The patterns of the research publications in this field were mapped to determine journals, countries, institutions, co-cited authors, co-cited references, research topics, research trends, and emerging areas of research on neurotransmitters and tumor progression.

## Materials and Methods

### Data Sources and Search Strategies

A literature search was conducted using the Web of Science Core Collection (WoSCC) database on August 1, 2021, to reduce bias incurred by database updating. The search strategy employed was as follows: TI = (“neurotransmitter” or “neurotransmitter receptor” or “5-HT” or “Serotonin” or “Cholinergic” or “Ach” or “Muscarinic acetylcholine receptor” or “GABA” or “gamma-aminobutyric acid” or “histamine” or “glycine or glutamate” or “NMDA” or AMPA” or “aspartic acid” or “dopamine” or “adrenergic” or “norepinephrine” or epinephrine” or “Neurokinin”) AND TS = (“tum*r” or “neoplasm” or “cancer” or “carcinoma”) NOT TS = (“non-cancer” or “chronic pain”) AND TS = (“prognos*s” or “outcome” or “recurrence” or “overall survival” or “recurrence free survival” or “relapse-free survival” or “proliferation” or “invasion” or “metastas*s”) NOT TI = (“guideline” or “recommendation” or “consensus” or “case report” or “meta” or “review”) AND Language = English. Document Type was set to include “Articles” only from 2001 to 2021. After the primary data search, two researchers (Y Shi and J Luo) screened all manuscripts individually to ensure that they were relevant to the subject of this study.

### Bibliometric Online Platform Analysis

Web of Science (https://wcs.webofknowledge.com) was used to analyze the search results and plot a histogram showing the publication trend. Then, the WoSCC data were converted to UTF-8 format and imported into the Online Analysis Platform of Bibliometrics (http://bibliometric.com/), we chose “total literature analysis” option for different countries’ publication trends analysis and “partnership analysis” option for intercountry/regional analysis, respectively.

### CiteSpace Software Analysis

Full records and cited references of these publications were downloaded from the WoSCC database, saved in.TXT format, and then imported into the CiteSpace software V5.6R5 SE, 64 bits (Drexel University, Philadelphia, PA, USA) using the following settings: Time slicing from January 2001 to June 2021 at 1 year per slice. The selection uses a modified g-index in each slice: k = 25. For interinstitutional analysis, “Institution” was chosen in the Node Types parameter area, and the remaining settings were all the default values. For co-authorship network analysis, “Cited-author” was chosen from the Node Types as after importing data into CiteSpace. For document co-citation, the related parameters were set as the following: choosing “References” as the Node Type, choosing “Cosine” to calculate relationship strength, and choosing “Pathfinder” and “Pruning the merged network” for Pruning parameters area to simplify the network and highlight its important structural features (10). For keywords and references burst detection, “Keywords” and “References” were chosen for Node Type, respectively. After removing keywords with little significance (like cells, mice, etc.), the top 20 keywords with the strongest citation bursts were identified and presented using Microsoft Excel 2019. The references with the strongest citation bursts were displayed without deletion.

## Results

### Quantity and Trend Analysis of Published Papers

**Figure 1:**
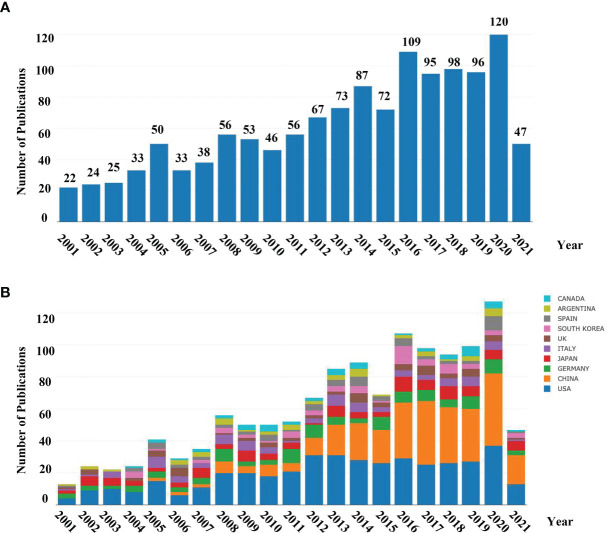
**(A)** Number of annual research publications and growth trends on the topic of neurotransmitters and tumor progression from 2001 to the first half of 2021, export of results from the Online Analysis Platform of Literature Metrology (http://biblimetric.com); **(B)** Number of annual publications and growth trends of the top 10 countries/regions on research in neurotransmitters and tumor progression from 2001 to 2021, export of results from the Online Analysis Platform of Literature Metrology (http://bibliometric.com). Bar chart reflects number of online articles online per year.

A total of 1310 publications met the inclusion criteria using our search strategy. The number of articles actually published each year was calculated using Online Analysis Platform of Bibliometrics (http://bibliometric.com/) (**Figure 1A**). The early stage (2000–2010) saw fluctuations in the number of publications above or below 50, with exceptions in 2005, 2008, and 2009. The number of papers published on the subject reached a peak in 2020, indicating that *neurotransmitters and cancer progression* have become a research hotspot and has captured global research attention.

To identify the countries/regions leading the research in the field, further analysis of publications in different countries and regions was conducted. The bar chart (**Figure 1B**) presents the top 10 countries/regions in terms of the total number of published articles in the past 20 years. Based on the number of publications and still increasing steadily, the USA was identified as a groundbreaker in the field. We also found that the annual publications in China have been increasing rapidly, outstripping the USA from early 2016.

### Journal Analysis

The WoSCC search showed that the 1310 papers included in the current analysis were published in 562 different journals over the last 20.5 years since 2001. Bibliometrics online analysis was used to analyze the influence of journals. The top 10 most cited journals are listed in **Supplementary Table 1**, among which, seven publishers are located in the USA, while the other three are located in Switzerland, Greece, and Netherlands, respectively. *Cancer Research*, which demonstrated the highest number of total citations (220) with an IF of 12.701, ranked first in the research field of the neurotransmitters and tumor progression.

### Analysis of Intercountry/Regional and Interinstitutional Cooperation

To determine the research institutional and interinstitutional cooperation in neurotransmitters and cancer research, we performed intercountry/regional and interinstitutional analyses using CiteSpace. After removing duplicate entries, 1275 published articles, 1 book chapter, 10 early-access articles, and 24 proceedings papers were identified, among which, 1285 (1275 published articles + 10 early-access articles) were included in the final analysis.

**Figure 2:**
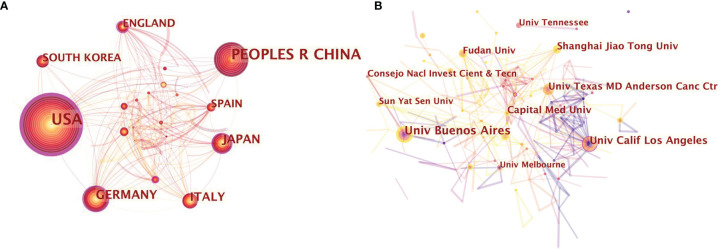
**(A)** Citespace network map of 68 countries involved in neurotransmitters and tumor progression research; **(B)** Citespace network map of institutions involved in neurotransmitters and tumor progression research. Each circle represents an institution. Size of circle is positively correlated with the number of articles published by institutions, and links between two circles represents a collaboration between two institutions on the same article. Line thickness is positively correlated with frequency of collaborations. Top 10 institutions with the most publications are shown. (the University of Buenos Aires, the University of California Los Angeles, the University of Texas MD Anderson Cancer Center, Shanghai Jiao Tong University, Fudan University, the University of Tennessee, Sun-Yat Sen University, Capital Medical University, the University of Melbourne, Consejo Nacl Investigation Cientificas & Tecn). Timespan: 2001-2021; Slice length=1.

Results of the intercountry/regional cooperation suggested that 68 countries have established partnerships, with 239 links among one other. USA and China possessed the best partnerships in this area. However, China showed less international cooperation than the USA (**Figure 2A**).

The top 10 most productive institutions are presented in **Figure 2B**. The size of the concentric circles signifies the number of publications, and the institution with more published articles tends to present larger concentric circles. Links between two institutions means they have jointly published articles. The boldness of lines indicates the strength of their cooperation. Collaborative relationship analysis among the different institutions yielded 534 nodes and 601 links. Institutions located in China and USA make up a substantial amount of the total. The University of Buenos Aires from Argentina was the most prolific institution. The second and the third productive institutions, the University of California Los Angeles and the University of Texas MD Anderson Cancer Center, were both located in the USA, followed by a Chinese institution, Shanghai Jiao Tong University.

### Author and Document Co-Citation Analysis

**Figure 3:**
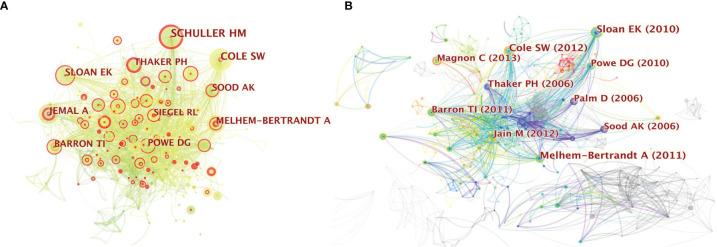
**(A)** Citespace network of co-cited authorship in the field of neurotransmitters and tumor progression research. Each circle represents one author. Size of circle is positively correlated with cited counts of the authors, and links between two circles represents a collaboration between two authors on the same article. Line thickness is positively correlated with frequency of collaborations. Top 10 most-cited authors are shown. Timespan: 2001-2021; Slice length=1; **(B)** Citespace co-citation map of 39677 references on neurotransmitters and tumor progression research, filter option showing the largest connected component only. Each circle represents a reference. Size of circle is positively correlated with frequency of citations, and links between two circles represent two references that were cited in the same article. Year and first author of the top 10 most-cited publications are shown. Timespan: 2001-2021; Slice length=1.

Co-citation analysis can reveal the research trends on *neurotransmitters and tumor progression*. We performed cited-author and cited-references analyses to find the top 10 most cited authors and references, which can provide important clues. A co-citation relationship among authors is established when two (or more) authors are cited in one or more subsequent papers at the same time. We can obtain a clear picture of core authors and their contributions to a certain field by analyzing the authors’ co-cited networks, the strength of which indicates the degree of participation of the authors. CiteSpace was used to analyze the 1285 original articles and 39677 valid and distinct references obtained from them to identify the top 10 most cited authors and references on *neurotransmitters and tumor progression*. In the author co-citation analysis, 858 nodes and 2775 links were obtained. The node size was positively associated with the cited counts of the authors, and the thickness of the lines between every two nodes represented the frequency of being co-cited between those two authors. The top 10 most-cited authors in this research area are shown in **Figure 3A**. HM Schuller from the University of Tennessee was the most-cited author and has been cited 88 times in 2007. SW Cole from the University of California Los Angeles has been cited 82 times in 2011 and thus ranks the second highest in most-cited authors. The other eight major research teams are also presented in **Figure 3A** (EK Sloan from the University of Melbourne, A Melhem-Bertrandt from the University of Texas, AK Sood from the MD Anderson Cancer Center, A Jemal and RL Siegel from American Cancer Society, PH Thaker from Washington University, DG Powe from Nottingham Trent University, and TI Barron from St. James’ Hospital in Ireland).

As for the document co-citation analysis, the year and the first author of the top 10 most-cited publications are shown in **Figure 3B**. The size of the circle is positively correlated with the frequency of citations, whereas the thickness of the lines between every two nodes represents the co-occurrence of citations. The details of these 10 articles are listed in **Supplementary Table 2**.

Since studies are usually cited to bolster the conceptions of the authors, a high citation frequency would reflect that the reference has made wide contributions in the field with highly proven peer recognition. Interestingly, the top 10 most-cited studies were mainly on the stress-correlated adrenaline system (11–20). Furthermore, breast cancer (BC) and prostate cancer have become the focus in this area, with the frequency of 6 for BC and 3 for prostate cancer. For example, the highest-ranking article published in the *Journal of Clinical Oncology* in 2011 (11) demonstrated that beta-blocker intake was associated with improved relapse-free survival (RFS) in 1413 patients with BC [hazard ratio (HR), 0.52; 95% confidence interval (CI), 0.31–0.88; P = 0.008] and in 377 patients with triple-negative breast cancer (TNBC) (HR, 0.30; 95% CI, 0.10–0.87; P = 0.027), indicating the protective function of beta-blockers. These were consistent with the results of the article with the second highest citation, released by *Cancer Research (* 12), which discussed that stress-induced neuroendocrine activation induced a 30-fold increase in metastasis to distant tissues. Accordingly, treatment with the beta-antagonist propranolol inhibited tumor spread.

### Clustered Network in Co-Analysis

**Figure 4:**
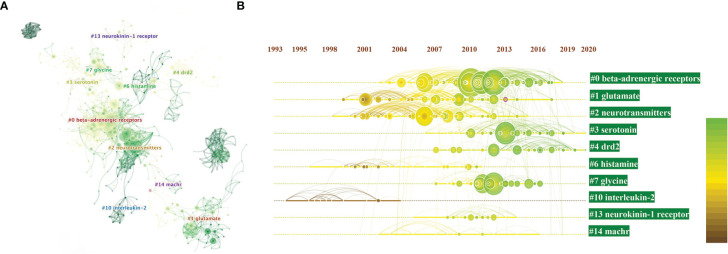
**(A)** Clustered networks of co-citation status of the investigated 39677 references and the 1285 citing articles *via* CiteSpace. The top 10 largest clusters of citing articles are shown; **(B)** Timeline view of the top 10 largest clusters of citing articles in the field of neurotransmitters and tumor progression research. Machr also known as nAchRs. Right side = cluster labels.

Next, we performed clustered network analysis to conduct a more in-depth study of those co-citations. If two publications have many common references, they are inclined to be homogenous. Based on this logic, we could divide 1285 articles into several clusters. After filter disposal by choosing “show the largest connected component only” node (which could explain why the displayed clustering numbers are not continuous), 10 major clusters generated from the co-citation networks of 39677 references cited by 1285 publications were identified. Cluster labels were salient noun phrases extracted from keywords using least square filtering (LSR) algorithm, including #0 beta-adrenergic receptors, #1 glutamate, #2 neurotransmitters, #3 serotonin, #4 DRD2, #6 histamine, #7 glycine, #10 inerleukin-2, #13 neurokinin receptor-1, and #14 nAchRs. The number of cluster tags as reversely correlated with the number of articles for each cluster included. Simply put, the cluster of #0 contains the largest number of articles (**Figure 4A**).

A timeline view of the distinct co-citations is shown in **Figure 4B** to present all the cited literature more clearly. The bold timeline indicates that the clustering topic was a hotspot during this period. Citation tree-rings with different sizes on the timeline represent some key articles with a high citation frequency.

We found that in the research on neurotransmitters and tumor progression, beta-adrenergic receptors (β-AR) has been a hot topic since 2004, reaching its peak moment in 2010. Studies on glutamate first appeared in 1999 and made a robust comeback in 2009. Serotonin was an emerging research field in 2013 and has attracted increasing attention recently. To our interest, inerleukin-2 (IL-2) is the only cytokine in the 10 major clusters post filtration, indicating that IL-2 may be an important cytokine related to the mechanism underlying the influence of neurotransmitters on tumor progression.

### Research Trend Analysis *via* Burst Detection With Keywords and References

**Figure 5:**
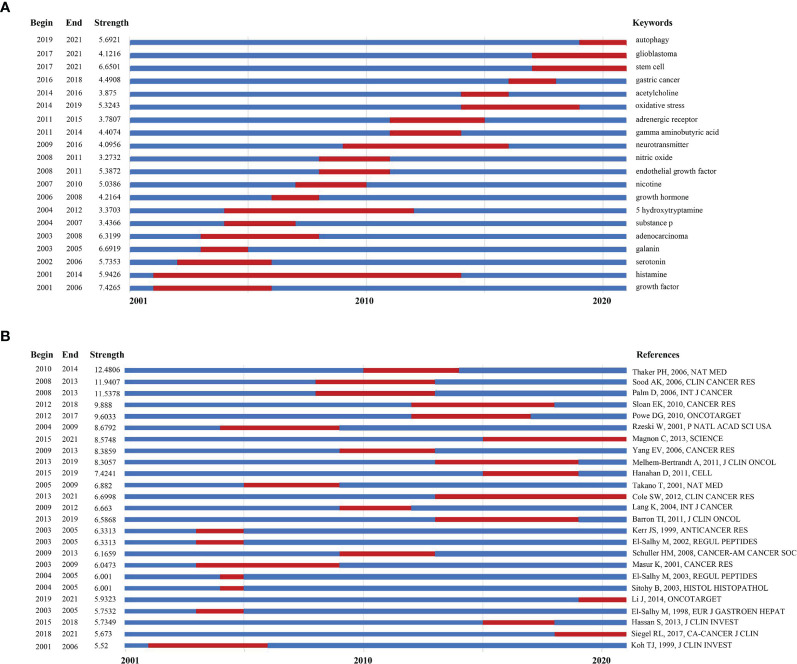
**(A)** Keywords with the strongest burst strength of the 1285 citing articles on neurotransmitters and tumor progression research between 2001 and 2021. Keywords marked in red indicates a sudden increase in the usage frequency of this keyword during that period. Blue represents a relatively unpopular time period; **(B)** References with the strongest burst strength of the 39677 references on neurotransmitters and tumor progression research between 2001 and 2021. References marked in red indicates a sudden increase in cited frequency of this article during that period. Blue represents a relatively unpopular time period.

Keywords burst detection was applied to acquire a quick glimpse of future research trends (**Figure 5A**). The red line indicates that the use of a keyword increased suddenly during the relevant period. In contrast, a blue line means relative unpopularity. The keywords burst detection identified histamine as a hot topic during 2001–2014, and researchers in this area have given increasing attention on mechanisms related to stem cells or autophagy (21); moreover, gastric cancer and glioblastoma have been the top two focal points of recent studies.

The top 25 references with the strongest citation bursts were also identified *via* a document co-citation strength analysis, which is another method for determining research trends (**Figure 5B**). The article with the strongest citation burst was published in Nature Medicine in 2006, which demonstrated that in ovarian cancer (22), beta-2-adrenergic receptor (β2-AR) activation mediated by chronic stress promotes tumor growth and angiogenesis, consistent with the results of the study by Sood et al. (18). While β2-AR has always been a hotspot in this research area since the early 2010s (14), dopamine receptor is also likely to be an emerging target for cancer development. An article aiming to clarify the role of dopamine receptor D2 (DRD2) in glioblastoma progression was cited consistently for 2 years since 2019 (23). The details of these 25 articles are listed in **Supplementary Table 3**.

## Discussion

This study visualized the citation analysis of research articles on *neurotransmitters and tumor progression* from 2001 to 2021. Our search strategy provides a comprehensive picture of neurotransmitters and their receptors in different descriptions. The number of published articles on this topic rapidly increased after 2016 and reached 120 articles per year in 2020. Using an online bibliometric analysis platform and CiteSpace, our study analyzed the publications on neurotransmitters and tumor progression from multiple dimensions and showed a systematic view for understanding this field over the past 20 years. These findings can guide future studies. Researchers new to this field can easily gain useful and relevant information from our bibliometric analysis.

USA, China, and Germany are the top three countries that have focused research on *neurotransmitters and tumor progression*. Notably, by 2016, the number of articles from China had outstripped those from the USA, making China the most prolific country in the field. The top three most fruitful research institutions were located in Argentina and the USA, respectively, followed by Shanghai Jiao Tong University from China. However, Chinese researchers were not in the top 10 most-cited authors, indicating that the quality and influence of their research can still improve. Interestingly, the top 10 most-cited studies were mainly on the stress-correlated adrenaline system, and BC and prostate cancer are the most commonly studied diseases in this area.

The timeline view of the 39677 related references and burst detection of keywords and references both indicated the research trends on neurotransmitters and tumor progression. We found that β2-AR, which is strongly linked with stress, was the most frequently studied neurotransmitter receptor since the early 2010s. Interestingly, chronic stress and acute stress seem to play distinct roles in tumor development and progression. While blocking chronic adrenergic signaling with beta-blockers shows protective potential in patients with cancer, activating acute adrenergic signaling through exercise also seems beneficial (24). Other neurotransmitter receptors, like dopamine receptors, have been increasingly studied since 2019. Dopamine can inhibit liver cancer cell growth and metastasis by activating dopamine receptors (25). Discrepant studies have also shown that patients with liver cancer with a high dopamine receptor D1 (DRD1) expression had worse prognosis, and the usage of the DRD1-specific antagonist SCH23390 significantly inhibited cell invasion and migration *in vitro* and tumor growth *in vivo (* 26). IL-2, the only cytokine appeared in the 10 largest clusters of citing articles, seems to exert a synergistic effect with antihistamine treatments in patients with acute myeloid leukemia and other cancers (27, 28). When administered together, antihistamines could enables the activation of T cells and NK cells by IL-2, resulting in the killing of tumor cells of various cancers (29), indicating a close connection between IL-2 and histamine signaling pathway in tumor development. The clustered network analysis also revealed that gastric cancer and glioblastoma have increasingly gained attention and have become the focal points of recent studies.

The explored connection between the nervous system and tumor tissue is not surprising, as they share a reciprocal impact. On the one hand, nerve fibers or neurotransmitters in tumor tissues not only can act on fibroblasts in the microenvironment by modulating the extracellular matrix synthesis or shaping synaptic-like connections with tumor cells (30) but also has a direct effect on immune cells, thereby regulating the infiltration of immune cells into tumors (7, 8). On the other hand, the invasion of the surrounding nerves by cancer cells also provides a route for metastasis and promotes tumorigenesis *via* the crosstalk between neuron cells and tumor cells (31, 32). A recent study has shown that perineural invasion (PNI) contributed to the immune-suppressive microenvironment in pancreatic ductal adenocarcinoma through the hyperactivation of PNI-associated cholinergic signaling (33).

The perioperative time period is a dangerous window for tumor metastasis, during which anesthesia contributes a significant part (34). Whether anesthetics or different anesthetic techniques influence the prognosis of patients with cancer has been a topic of interest recently (35–39). First, patients with cancer show higher stress and anxiety levels than other patients (40, 41). The use of anesthetics reduces patients’ pain and relieves the stress caused by the surgery. Second, anesthetics themselves would directly affect the malignancy of tumor cells. Most fundamental studies focusing on anesthetics have proven that propofol, midazolam, and local anesthetics exert potential anti-cancer properties, whereas inhalants and opioids promote cancer development. The potential mechanisms underlying the effects of anesthetics are more or less associated with neurotransmitters or their receptors because the primary targets of many anesthetics are neurotransmitter receptors.

Among these, GABA receptors are most closely associated with anesthesia and sedation. Propofol, inhaled anesthetics, etomidate, and benzodiazepine sedatives can all act on GABA receptors. In a mouse model of lung metastasis from colon cancer, Xie et al. (42) found that propofol could downregulate the expression of the ubiquitination regulatory protein TRIM21 by activating GABAA receptors on the tumor surface, thereby upregulating Src protein and increasing the adhesion of tumor cells to vascular endothelial cells, which ultimately promotes distal metastasis of tumor cells to the lung. However, strong data have been presented that support the inhibitive impact of propofol on tumor development by acting on GABA receptors. In glioma studies, propofol, etomidate, and diazepam modulated GABA receptor function and inhibited glioma cell proliferation by inducing cell cycle arrest (43, 44). The involvement of NMDA receptors in propofol-induced inhibition of tumor growth and metastasis has also been reported in other studies (45, 46). Besides GABA and NMDA, the connection among anesthetics, other neurotransmitters, and tumor progression has also been demonstrated. Dexmedetomidine (Dex) is an alpha-2-adrenergic receptor (α2-AR) agonist, also a sedative drug commonly used in clinical anesthesia. Studies have suggested that the perioperative use of Dex or α2-AR activation promotes tumor cell proliferation (47, 48). Wang et al. (49) demonstrated that Dex could promote tumor cell migration by activating the α2-AR/STAT3 pathway and the secretion of the exosomal protein TMPRSS2 in breast cancer. We also previously found that postoperative serum from patients administered with Dex during surgery promoted BC cell proliferation, migration, and invasion, indicating that Dex worsens the prognosis of patients with BC (50). Additionally, there is a paucity of studies on the prognostic effects of muscle relaxants, a blocker of the binding between Ach and nAchRs, in patients with cancer. A few studies have found that cis-atracurium has some anti-colon cancer effects *in vitro*, however, the link between its anti-cancer effects and nAchRs is unclear (51, 52).

This study has several limitations. First, the data were retrieved from a single database. Second, only articles with English keywords or abstracts in the database were considered in our analysis, and articles in other languages were thus excluded. Third, bibliometrics studies are a type of quantitative analysis of scholarly publications that can only be conducted with publications in journals that are cited and indexed but not unpublished studies or publications in non-indexed journals, dissertations, books, or government reports. In future studies, we may use multimethod evaluations to gain a more in-depth understanding of this research field.

The findings from this bibliometric study provided insights into research trends on neurotransmitters and tumor progression in the past 20 years. The study provides new directions and ideas for clinical tumor treatment by targeting neurotransmitters and their receptors and for the optimization of anesthetic techniques and medications in patients with cancer. Priority should be given to anesthetics that may be beneficial to the prognosis of patients with cancer. Furthermore, the effects of perioperative stress, anxiety, and depression and other psychiatric factors on the nervous system should be fully considered to reduce risk factors that may accelerate tumor progression.

## Author Contributions

Conception and design: YS. Administrative support: HZ, WY, DS, and JT. Collection and assembly of data: YS and JL. Data analysis and interpretation: XW, YZ, and HZ. Manuscript writing: All authors. Final approval of manuscript: All authors.

## Funding

This study received financial support from the Shanghai Science and Technology Committee Foundation (grant number 19ZR1430600), Clinical Research Plan of SHDC (grant number SHDC2020CR4062), Shanghai Municipal Commission of Health and Family Planning (grant number 201840241), Shanghai Pudong New Area Municipal Commission of Health and Family Planning Funding (grant number PWZxq2017-06), Shanghai Municipal Key Clinical Specialty (grant number shslczdzk03601 to WY) and Shanghai Engineering Research Center of Peri-operative Organ Support and Function Preservation (grant number 20DZ2254200).

## Conflict of Interest

The authors declare that the research was conducted in the absence of any commercial or financial relationships that could be construed as a potential conflict of interest.

## Publisher’s Note

All claims expressed in this article are solely those of the authors and do not necessarily represent those of their affiliated organizations, or those of the publisher, the editors and the reviewers. Any product that may be evaluated in this article, or claim that may be made by its manufacturer, is not guaranteed or endorsed by the publisher.

## Abbreviations

WHO, World Health Organization; WoSCC, Web of Science Core Collection; USA, United States of America; Ach, Acetylcholine; nAchRs, Nicotinic acetylcholine receptors; GABA, Gamma-aminobutyric acid; NMDA, N-methyl-D-aspartic acid receptor; NE, Norepinephrine; E, Epinephrine; NPY, Neuropeptide Y; HR, Hazard ratio; CI, Confidence interval; BC, Breast cancer; TNBC, Triple negative breast cancer; IL-2, Inerleukin-2; B-AR, Beta-adrenergic receptors; B2-AR, Beta2-adrenergic receptors; α2-AR, Alfa2- adrenergic receptor; DRD2, Dopamine receptor D2; DRD1, Dopamine receptor D1; Dex, Dexmedetomidine; HIF-1, Hypoxia-inducible factor 1; TI, Title; TS, Topical subject; IF, Impact factors; LSR, least square filtering.
