# Supplementary material for: Emerging Trends on the Correlation Between Neurotransmitters and Tumor Progression in the Last 20 Years: A Bibliometric Analysis via CiteSpace
Source: Front Oncol. 2022 Feb 24;12:800499. doi: 10.3389/fonc.2022.800499 (PMC8907850; doi:10.3389/fonc.2022.800499)
Supplement: Supplementary file 1 [file Table_1.docx]

**Supplemental Table 1. The top 10 most active journals that published articles in anesthesia and cancer research (sorted by total citation)**

| Rank | Journal Title | Frequency | Total citations | Average citation per paper | Impact factor  (2020) | Country | JCR |
| --- | --- | --- | --- | --- | --- | --- | --- |
| 1 | Cancer Research | 23 | 220 | 9.57 | 12.701 | USA | Q1 |
| 2 | Clinical Cancer Research | 20 | 148 | 7.40 | 12.531 | USA | Q1 |
| 3 | International Journal of Cancer | 16 | 136 | 8.5 | 7.396 | Switzerland | Q2 |
| 4 | Breast Cancer Research and Treatment | 10 | 107 | 10.7 | 4.872 | Netherlands | Q2 |
| 5 | Brain Behavior and Immunity | 14 | 84 | 6.00 | 7.217 | USA | Q2 |
| 6 | Proceedings of the National Academy of Sciences of the United States of America | 7 | 82 | 11.71 | 11.205 | USA | Q1 |
| 7 | Oncology Reports | 22 | 80 | 3.64 | 3.906 | Greece | Q3 |
| 8 | Cancer Biology & Therapy | 9 | 75 | 8.33 | 4.742 | USA | / |
| 9 | Plos One | 26 | 73 | 2.81 | 3.240 | USA | / |
| 10 | Nature Medicine | 4 | 56 | 14 | 53.440 | USA | Q1 |
