# Supplementary material for: Emerging Trends on the Correlation Between Neurotransmitters and Tumor Progression in the Last 20 Years: A Bibliometric Analysis via CiteSpace
Source: Front Oncol. 2022 Feb 24;12:800499. doi: 10.3389/fonc.2022.800499 (PMC8907850; doi:10.3389/fonc.2022.800499)
Supplement: Supplementary file 2 [file Table_2.docx]

**Supplemental Table 2. The top 10 high-cited papers in neurotransmitter and cancer research during 2001 to 2021**

| Rank | Title | First Author | Journal | Year | Cited Frequency | DOI |
| --- | --- | --- | --- | --- | --- | --- |
| 1 | Beta-blocker use is associated with improved relapse-free survival in patients with triple-negative breast cancer. | Melhem-Bertrandt A | J CLIN ONCOL | 2011 | 28 | 10.1200/JCO.2010.33.4441 |
| 2 | The sympathetic nervous system induces a metastatic switch in primary breast cancer. | Sloan EK | Cancer Research | 2010 | 27 | 10.1158/0008-5472.CAN-10-0522 |
| 3 | Beta-blocker drug therapy reduces secondary cancer formation in breast cancer and improves cancer specific survival. | Powe DG | Oncotarget | 2010 | 24 | 10.18632/oncotarget.101009 |
| 4 | Molecular pathways: beta-adrenergic signaling in cancer. | Cole SW | Clinical Cancer Research | 2012 | 24 | 10.1158/1078-0432.CCR-11-0641 |
| 5 | Beta blockers and breast cancer mortality: a population- based study | Barron TI | J CLIN ONCOL | 2011 | 23 | 10.1200/JCO.2010.33.5422 |
| 6 | Metabolite profiling identifies a key role for glycine in rapid cancer cell proliferation. | Jain M | Science | 2013 | 21 | 10.1126/science.1218595 |
| 7 | Autonomic nerve development contributes to prostate cancer progression. | Magnon C | Science | 2013 | 17 | 10.1126/science.1236361 |
| 8 | Stress hormone-mediated invasion of ovarian cancer cells. | Sood AK | Clinical Cancer Research | 2006 | 16 | 10.1158/1078-0432.CCR-05-1698 |
| 9 | The norepinephrine-driven metastasis development of PC-3 human prostate cancer cells in BALB/c nude mice is inhibited by beta-blockers. | Palm D | Internation Journal of Cancer | 2006 | 15 | 10.1002/ijc.21723 |
| 10 | Behavioral stress accelerates prostate cancer development in mice. | Hassan S | J CLIN INVEST | 2013 | 15 | 10.1172/JCI63324 |
