# Supplementary material for: Emerging Trends on the Correlation Between Neurotransmitters and Tumor Progression in the Last 20 Years: A Bibliometric Analysis via CiteSpace
Source: Front Oncol. 2022 Feb 24;12:800499. doi: 10.3389/fonc.2022.800499 (PMC8907850; doi:10.3389/fonc.2022.800499)
Supplement: Supplementary file 3 [file Table_3.docx]

**Supplemental Table 3. The top 25 references with the strongest citation bursts**

| Rank | Title | DOI | Year | Begin | End | Strength |
| --- | --- | --- | --- | --- | --- | --- |
| 1 | Chronic stress promotes tumor growth and angiogenesis in a mouse model of ovarian carcinoma | 10.1038/nm1447 | 2006 | 2010 | 2014 | 12.4806 |
| 2 | Stress Hormone–Mediated Invasion of Ovarian Cancer Cells | 10.1158/1078-0432.CCR-05-1698 | 2006 | 2008 | 2013 | 11.9407 |
| 3 | The norepinephrine-driven metastasis development of PC-3 human prostate cancer cells in BALB/c nude mice | 10.1002/ijc.21723 | 2006 | 2008 | 2013 | 11.5378 |
| 4 | The Sympathetic Nervous System Induces a Metastatic Switch in Primary Breast Cancer | 10.1158/0008-5472.CAN-10-0522 | 2010 | 2012 | 2018 | 9.888 |
| 5 | Beta-Blocker Drug Therapy Reduces Secondary Cancer Formation in Breast Cancer and Improves Cancer Specific Survival | 10.18632/oncotarget.197 | 2010 | 2012 | 2017 | 9.6033 |
| 6 | Glutamate antagonists limit tumor growth | 10.1073/pnas.091113598 | 2001 | 2004 | 2009 | 8.6972 |
| 7 | Autonomic Nerve Development Contributes to Prostate Cancer Progression | 10.1126/science.1236361 | 2013 | 2015 | 2021 | 8.5748 |
| 8 | Norepinephrine Up-regulates the Expression of Vascular Endothelial Growth Factor, Matrix Metalloproteinase (MMP)-2, and MMP-9 in Nasopharyngeal Carcinoma Tumor Cells | 10.1158/0008-5472.CAN-06-2496 | 2006 | 2009 | 2013 | 8.3859 |
| 9 | Beta-Blocker Use Is Associated With Improved Relapse-Free Survival in Patients With Triple-Negative Breast Cancer | 10.1200/JCO.2010.33.4441 | 2011 | 2013 | 2019 | 8.3057 |
| 10 | Hallmarks of Cancer: The Next Generation | 10.1016/j.cell.2011.02.013 | 2011 | 2015 | 2019 | 7.4241 |
| 11 | Glutamate release promotes growth of malignant gliomas | 10.1038/nm0901-1010 | 2001 | 2005 | 2009 | 6.882 |
| 12 | Molecular Pathways: Beta-Adrenergic Signaling in Cancer | 10.1158/1078-0432.CCR-11-0641 | 2012 | 2013 | 2021 | 6.6998 |
| 13 | Induction of a metastatogenic tumor cell type by neurotransmitters and its pharmacological inhibition by established drugs | 10.1002/ijc.20410 | 2004 | 2009 | 2012 | 6.663 |
| 14 | Beta Blockers and Breast Cancer Mortality: A Population- Based Study | 10.1200/JCO.2010.33.5422 | 2011 | 2013 | 2019 | 6.5868 |
| 15 | Novel small molecule alpha v integrin antagonists: comparative anti-cancer efficacy with known angiogenesis inhibitors | / | 2001 | 2003 | 2005 | 6.3313 |
| 16 | Triple therapy with octreotide, galanin and serotonin induces necrosis and increases apoptosis of a rat colon carcinoma - ScienceDirect | 10.1016/S0167-0115(02)00106-4 | 2002 | 2003 | 2005 | 6.3313 |
| 17 | GABAB receptor is a novel drug target for pancreatic cancer | 10.1002/cncr.23231 | 2008 | 2009 | 2013 | 6.1659 |
| 18 | Norepinephrine-induced migration of SW 480 colon carcinoma cells is inhibited by beta-blockers | / | 2001 | 2003 | 2009 | 6.0473 |
| 19 | Triple therapy with octreotide, galanin, and serotonin reduces the size and blood vessel density and increases apoptosis of a rat colon carcinoma. | 10.1016/s0167-0115(02)00280-X | 2003 | 2004 | 2005 | 6.001 |
| 20 | A comparison between double and triple therapies of octreotide, galanin and serotonin on a rat colon carcinoma | 10.14670/HH-18.103 | 2003 | 2004 | 2005 | 6.001 |
| 21 | Genome-wide shRNA screen revealed integrated mitogenic signaling between dopamine receptor D2 (DRD2) and epidermal growth factor receptor (EGFR) in glioblastoma | 10.18632/oncotarget.1801 | 2014 | 2019 | 2021 | 5.9323 |
| 22 | Colonic endocrine cells in patients with carcinoma of the colon. | 10.1097/00042737-199806000-00015 | 2001 | 2003 | 2005 | 5.7532 |
| 23 | Behavioral stress accelerates prostate cancer development in mice | 10.1172/JCI63324 | 2013 | 2015 | 2018 | 5.7349 |
| 24 | Cancer Statistics, 2017 | 10.3322/caac.21387 | 2017 | 2018 | 2021 | 5.673 |
| 25 | Overexpression of glycine-extended gastrin in transgenic mice results in increased colonic proliferation | 10.1172/JCI4910 | 2001 | 2018 | 2021 | 5.52 |
